# Linking AIM2 Inflammasome Activation, Mitochondrial Dysfunction and Chronic Inflammation in Ankylosing Spondylitis

**DOI:** 10.3390/cells14231923

**Published:** 2025-12-03

**Authors:** Catalina Alina Boengiu, Andreea-Lili Barbulescu, Cristiana Cerasella Dragomirescu, Ana-Maria Buga, Adina Andreea Mirea

**Affiliations:** 1 Doctoral School, University of Medicine and Pharmacy of Craiova, 200349 Craiova, Romania; 2 Department of Pharmacology, University of Medicine and Pharmacy of Craiova, 200638 Craiova, Romania; 3 Department of Microbiology, Carol Davila University of Medicine and Pharmacy, 020021 Bucharest, Romania; 4 Department of Biochemistry, University of Medicine and Pharmacy of Craiova, 200638 Craiova, Romania; 5 Department of Oro-Dental Prevention, University of Medicine and Pharmacy of Craiova, 200638 Craiova, Romania

**Keywords:** AIM2 inflammasome, mitochondrial dysfunction, mtDNA, ankylosing spondylitis, autoimmunity, targeted therapy

## Abstract

The absent in melanoma 2 (AIM2) inflammasome is a cytosolic DNA sensor that links genomic instability, mitochondrial dysfunction, and chronic inflammation. Unlike the nucleotide-binding domain, leucine-rich repeat (NLR) family pyrin domain-containing protein 3 (NLRP3) inflammasome, AIM2 is activated directly by double-stranded Deoxyribonucleic Acid (dsDNA), including mitochondrial DNA (mtDNA) released under stress conditions. This positions AIM2 at the intersection of oxidative stress, impaired mitophagy, and innate immune dysregulation. Current therapies for ankylosis spondylitis (AS), such as anti-tumor necrosis factor (TNF), anti-interleukin 17 (IL-17), and Janus kinase (JAK) inhibitors, improve clinical outcomes; however, they do not address upstream mitochondrial dysfunction or DNA-driven inflammasome activation. By contrast, other inflammasomes, such as AIM2, remain comparatively less studied. Since autoimmune diseases, including AS, are frequently accompanied by uncontrolled innate immune responses to self-DNA, these findings provide a framework for comprehending the mechanisms of AIM2 activation and its interaction with inflammation, mitophagy, and oxidative stress. Here, we review the current evidence on AIM2 inflammasome involvement in AS pathogenesis and its potential as a therapeutic target. This approach offers new insight into disease control through re-establishing the balance between mitochondrial dysfunction and autoimmunity.

## 1. Introduction

Ankylosing spondylitis (AS) is a disease in which chronic inflammation, mitochondrial dysfunction, and innate immune activation converge. The disease etiology remains incompletely understood. The majority of AS patients are human leucocyte antigen-B27 (HLA-B27) positive, but this pattern is not directly associated with disease onset or progression, indicating that other mechanisms contribute to AS pathogenesis [1,2]. Nowadays, AS is recognized as having both autoinflammatory and autoimmune components.

The inflammasome regulates host immune responses by detecting pathogen-associated molecular patterns (PAMPs), danger-associated molecular patterns (DAMPs), and homeostasis-altering molecular processes (HAMPs) [3]. Upon activation, inflammasomes trigger caspase 1 (casp-1) activation, which mediates the proteolytic cleavage of pro-inflammatory cytokines into their mature, active forms (e.g., interleukin 1β-IL-1β, interleukin 18–IL-18) and initiates pyroptosis [4,5,6].

The concept of the inflammasomes was first proposed in 2002 by Martinon and colleagues [7], who described a caspase-activating cytosolic complex formed by NLR family pyrin domain-containing protein 1 (NLRP1), together with the pyrin (PYD) and caspase recruitment (CARD) domain-containing gene Pycard. The Pycard gene encodes apoptosis-associated speck-like protein containing a CARD (Apoptosis-Associated Speck-like protein -ASC), an adaptor protein that recruits and activates casp-1 and caspase-5 (casp-5), thereby promoting IL-1β and IL-18 production [7,8].

The origin of inflammasome research can be traced back to the discovery of “leukocytic pyrogen”, which marked the beginning of a new era in immunology focused on the molecular mediators of fever and inflammation. In the decades that followed, eleven other molecules were identified as key endogenous pyrogens, establishing their central role in innate immune defense [9].

**Figure 1:**
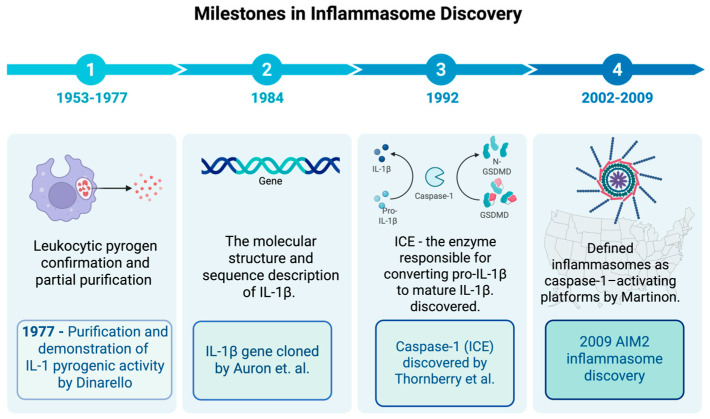
Timeline of inflammasome research milestones. Schematic timeline highlighting key discoveries in inflammasome biology. Early work between 1953 and 1954 focused on the confirmation and isolation of leukocytic pyrogen, while the purification of IL-1 as leukocytic pyrogen was performed by Dinarello and colleagues in 1977 [11]. In 1984, Auron and colleagues [12] cloned the IL-1β gene and revealed that it is produced as an inactive precursor requiring further proteolytic cleavage to become active, raising important questions about the mechanisms underlying this process. In 2002, Martinon et al. [7] introduced the concept of the inflammasome, detailing NLRP1 and ASC as core components. In the years that followed, the family of inflammasome sensors (NLRP3, AIM2, NLRC4, etc.) grew, and their roles in caspase-1 activation, IL-1β/IL-18 maturation, and pyroptosis became clearer [13]. In 2009, the AIM2 inflammasome was identified as a cytosolic DNA sensor. It was also shown to play a role in chronic inflammatory diseases. ASC stands for apoptosis-associated speck-like protein with a CARD, and NLR stands for nucleotide-binding oligomerization domain-like receptor. Created in BioRender. b, a. (accessed on 15 November 2025) https://BioRender.com/6kxwkgo.

Dinarello and colleagues later demonstrated the pyrogenic activity of purified human IL-1β in rabbits [10,11]. By 1984, the nucleotide sequence of IL-1β was determined using complementary DNA (cDNA) cloning, laying the foundation for understanding cytokine signaling pathways and paving the way for the later inflammasomes identification of inflammasomes as a key pathway for IL-1β maturation [12]. Since the first description of the inflammasome in 2002, subsequent studies have expanded the family of identified inflammasomes (e.g., NLRP3, AIM2, NLR family CARD domain containing 4-NLRC4) and clarified their roles in casp-1 activation, IL-1β/IL-18 maturation, and pyroptosis. These advances have firmly established inflammasomes as key innate immune sensors involved in the pathogenesis of chronic inflammatory, metabolic, and autoimmune diseases (Figure 1).

Building on these early discoveries, subsequent studies have focused on defining the molecular mechanisms of inflammasome activation and their role in disease. In the last decade, inflammasome research has progressed rapidly, establishing it as a first-line innate immune system sensor involved in the pathogenesis of many diseases (e.g., cardiovascular diseases, metabolic disorders, or cancer) [14,15,16,17]. Among inflammasomes, NLRP3 is the most studied innate immune sensor; its signaling network and mechanism of activation are well known. Although several recent reviews have focused on the NLPR3 inflammasome, the AIM2 inflammasome role remains comparatively underexplored [18,19,20,21,22,23].

The absent in melanoma 2 (AIM2) inflammasome is a cytosolic Deoxyribonucleic Acid (cDNA) sensor that links genomic instability, mitochondrial dysfunction, and chronic inflammation. Unlike the nucleotide-binding domain, the leucine-rich repeat (NLR) family pyrin domain-containing protein 3 (NLRP3), AIM2 is activated directly by double-stranded DNA (dsDNA), including mitochondrial DNA (mtDNA) released under stress conditions. This positions AIM2 at the intersection of oxidative stress, impaired mitophagy, and innate immune dysregulation) [24,25,26]. While the NLP3 remains the most studied inflammasome, AIM2 has received comparatively less attention in the context of autoinflammatory and autoimmune diseases. AIM2 is a distinct DNA-sensing inflammasome with a well-defined activation mechanism, and its ability to detect self-derived dsDNA provides an important framework for understanding how mitochondrial stress, impaired mitophagy, and oxidative damage can drive inflammatory responses.

Since dysregulated innate immune responses to self-DNA are a hallmark of autoimmune diseases such as lupus erythematosus, psoriasis, systemic sclerosis, and certain interferonopathies, AIM2 offers a crucial framework for comprehending the interactions between DNA sensing, inflammation, mitophagy, and oxidative stress. In this review, we provide an overview of the available data regarding the role of the AIM2 inflammasome in chronic autoimmune and autoinflammatory diseases, with emphasis on its contribution to the pathophysiology of ankylosing spondylitis (AS) and its potential as a therapeutic target. This perspective provides new insights into how reestablishing the equilibrium between autoimmunity and mitochondrial homeostasis could enhance disease control by fusing innate immune signaling with mitochondrial dysfunction.

## 2. Mechanistic Insights

### 2.1. Mitochondrial Dysfunction as Trigger of Inflammasome Activation

Uncontrolled inflammasome activation, mitochondrial dysfunction, and oxidative stress are tightly interconnected processes that amplify one another and contribute to chronic inflammation in autoimmune and autoinflammatory diseases, including AS. Mitochondria not only generate cellular energy but also act as key regulators of innate immunity. Under pathological conditions, such as autoimmune disorders, mitochondrial stress becomes both a driver and a consequence of inflammation. It can be triggered by multiple factors, including pro-inflammatory cytokine (e.g., Il-1β/IL-17, TNF-α, interferon gamma-IFN-γ) [27,28]; immune cell overactivation which increases metabolic demand and overwhelm mitochondrial capacity [29,30,31,32]; and impaired mitochondrial clearance (defective mitophagy), which leads to the accumulation of dysfunctional mitochondria that produce excessive reactive oxygen species (ROS) and mitochondrial DNA (mtDNA) [33,34,35].

Autoimmune diseases are frequently accompanied by increased oxidative stress, which triggers mitochondrial damage and further increases ROS production [36,37]. Damaged or dysfunctional mitochondria release oxidized mitochondrial DNA (mtDNA) into the cytosol, where it functions as a danger-associated molecular pattern (DAMP) capable of activating innate immune sensors such as AIM2 and cGAS sensors. This activation leads to downstream inflammasome activation and IL-1β and IL-18 production.

In addition to endogenous sources of cellular stress, such as mitochondrial ROS overproduction, defective mitophagy, and chronic inflammatory signaling, environmental triggers, including infections and exogenous toxins, can also induce oxidative damage to mitochondrial DNA (mtDNA). These mechanisms may contribute to the onset of autoimmunity in genetically susceptible individuals [24,25,38].

Together, these mechanisms demonstrate that mitochondria are not passive bystanders during inflammation; rather, they act as active modulators of innate immune signaling. Establishing this mitochondrial–inflammasome axis is essential for understanding how AIM2 senses cytosolic DNA and contributes to autoinflammatory pathology.

### 2.2. AIM2 Inflammasome Sensor Structure and Activation

This tight interplay between mitochondrial dysfunction and cytosolic DNA sensing activation provides a strong rationale for examining the AIM2 inflammasome, a key DNA sensor that links cytosolic DNA stress to inflammatory responses. AIM2 was initially identified as a tumor suppressor gene in murine models of colorectal cancer [39,40]; however, other studies revealed that AIM2 also participates in innate immune signaling as a cytosolic DNA sensor and a key regulator of inflammasome activation [26,41].

AIM2 belongs to the AIM2-like receptor (ALR) family. In humans, this family includes several DNA-sensing proteins, such as gamma-interferon-inducible protein 16 (IFI16), pyrin, hematopoietic interferon-inducible nuclear protein (HIN), IFIX (a HIN-containing domain protein), and the myeloid cell nuclear differentiation antigen (MNDA) [42,43]. Unlike the more extensively studied NLR family, ALRs are activated directly by double-stranded DNA through their HIN200 domain, without requiring transcriptional priming for activation.

AIM2 protein contains two functional domains: an N-terminal pyrin domain (PYD), which interacts with the adaptor ASC, and a C-terminal domain, hematopoietic interferon-inducible nuclear protein with a 200-amino acid repeat domain (HIN200), which binds dsDNA in a sequence-independent manner. Both domains are essential for AIM2 inflammasome assembly and activation of [42,43].

HIN200 domain has two oligonucleotide/oligosaccharide-binding folds architecture that enables it to recognize and bind to cytoplasmic or nuclear dsDNA derived from damaged host cells or exogenous sources (e.g., viral cells). In the resting state, the PYD domain (a member of the death domain superfamily) is masked by its HIN200 domain to prevent spontaneous activation. Upon binding dsDNA—typically fragments >80 base pairs—the HIN200 domain undergoes conformational changes that expose the PYD domain [44]. This allows AIM2 to recruit ASC through PYD–PYD interactions. Because AIM2 is constitutively expressed in many cell types [45], it does not require NF-κB–mediated priming and can respond rapidly to cytosolic DNA.

The canonical AIM2 inflammasome can be activated by both endogenous dsDNA (resulting from mitochondrial damage, e.g., oxidized mtDNA) and exogenous dsDNA (e.g., bacterial or viral DNA) [46,47]. Unlike NLRP3, which responds to diverse triggers such as ROS, ion efflux, extracellular adenosine triphosphate (ATP), uric acid, cholesterol, and lysosomal damage, AIM2 is highly specific for dsDNA, making it uniquely positioned to sense DNA damage–associated stress [48,49].

Upon activation, AIM2 exposes its PYD and recruits the adaptor ASC, which contains both a PYD and a CARD domain. Similarly to AIM2, ASC serves as an adaptor protein with two functional domains: its N-terminal PYD domain interacts with AIM2, while its C-terminal domain, CARD, recruits pro-casp 1 through CARD-CARD interactions, enabling casp one activation [43,50]. Active caspase-1 then cleaves gasdermin D (GSDMD) to initiate pyroptosis and processes pro-IL-1β and pro-IL-18 into their mature, inflammatory forms. The pyroptosis process is an inflammatory form of cell death mediated by gasdermin D (GSDMD) pore formation, which drives cellular lysis and promotes inflammation.

Beyond its canonical role in pyroptosis, AIM2 activation also contributes to PANoptosis, an integrated cell death program that combines pyroptosis, apoptosis, and necroptosis. This process integrates multiple cell-death pathways into a single integrated program, ensuring that cellular death can proceed even when one pathway is inhibited, through compensatory switching between pathways [51]. AIM2 serves as a key component of the AIM2–PANoptosome, facilitating cell death pathway switching under inflammatory stress [51,52,53].

Recent evidence indicates that AIM2 can also be indirectly primed. For example, Carrillo–Gálvez and colleagues demonstrated that AIM2 inflammasome activation can be modulated by indirect priming signals, such as lipopolysaccharide (LPS), which upregulates AIM2 expression and increases the sensor’s sensitivity to dsDNA [54]. They also reported that under highly inflammatory conditions, AIM2 can be indirectly activated by mtDNA released following NLRP3 activation, increased ROS production, and pyroptosis [54]. This study provides “in vitro” evidence that AIM2 expression in macrophages can be enhanced by innate immune signals (e.g., LPS presence in the absence of dsDNA), suggesting a potential second-step activation mechanism. For the future, these mechanisms must be further proved in more complex studies, such as “in vivo” studies designed for better translational potential. The AIM2 inflammasome is activated in a highly specific manner primarily by self-DNA or mtDNA accumulation; it differs from the NLRP3 inflammasome, which responds as a general danger sensor that drives inflammation in many disorders (e.g., cardiovascular and metabolic disorders).

### 2.3. Cytosolic dsDNA as Danger Signal

The cytoplasmic dsDNA contributors include immune or stromal cells undergoing to oxidative stress, mitochondrial injury, genomic instability, or defective DNA repair mechanisms [55,56,57,58]. Under physiological conditions, cells prevent cytosolic DNA accumulation through their own protective mechanisms, such as enzymatic cleavage by DNAse and cytosolic DNA exonuclease (e.g., three prime repair exonuclease 1-TREX1), as well as through autophagy-mediated clearance of damaged mitochondria. These mechanisms degrade self-DNA before it can trigger innate immune sensors [59,60,61,62]. However, when these clearance mechanisms are overwhelmed or inefficient, dsDNA accumulates and functions as a DAMPs that triggers early innate immune responses, accompanied by low-level inflammation.

In addition, one major source of dsDNA is the micronuclei, a small DNA-containing structure formed during genomic instability. Nowadays, it receives special attention since a causal relationship between micronuclei formation and immune activation has been identified [63,64,65,66]. Under normal conditions, micronuclei are removed by micronucleophagy, a protective process that prevents the exposure of genomic DNA to the cytosol. This process is essential for preventing genomic instability and inflammation [67]. The micronuclear envelope splits the genome and cytoplasmic compartment, thereby maintaining normal nucleocytoplasmic translocation, which is essential for cellular homeostasis [68]. When this envelope is disrupted, dsDNA is exposed in the cytoplasm and triggers innate immune signaling pathways, such as the cyclic GMP-AMP synthase (cGAS)- stimulator of interferon genes (STING) pathway [69] and the AIM2/ASC inflammasome [70]. Elevated micronuclei formation has been reported in inflammation and autoimmune diseases, including rheumatoid arthritis, systemic lupus erythematosus, and systemic sclerosis [71]. This evidence suggests that micronuclei may represent a mechanistic link between genomic instability, oxidative stress, and chronic inflammation [72].

### 2.4. Mitochondrial Dysfunction and AIM2 Activation in AS

Alongside nuclear-derived DNA, mitochondrial stress and defective quality control add another layer of danger signaling that can drive AIM2 inflammasome activation. Mitochondria are not only the cellular energetic hub, but also act as regulators of innate immunity. Mitochondrial dysfunction and inflammasome activation are closely interrelated and play an important role in the development of inflammation-related pathology. While inflammation is protective in the short term, prolonged inflammatory states can play a central role in disease development. The main players in this context are immune cells such as macrophages, lymphocytes, and plasma cells. In many autoimmune and autoinflammatory disorders, including AS, chronic inflammation is accompanied by increased pro-inflammatory cytokine production and oxidative stress. In addition, increased mitochondrial ROS (mtROS) promotes mitochondrial permeability transition pore (mPTP) formation and DNA damage, leading to mtDNA leakage into the cytosol, where it acts as a DAMP to trigger inflammasome activation (both AIM2 through dsDNA and via NFkB pathway) [73,74,75,76]. This process promotes caspase-1–dependent pyroptosis and shifts cellular death from apoptosis (caspase-3–mediated) to inflammatory pyroptosis (caspase-1–mediated). Activated caspase-1 cleaves GSDMD and promotes mPTP formation, reinforcing mitochondrial dysfunction and inflammation.

Other mitochondrial danger signals, such as cardiolipin exposure, may also indirectly drive AIM2 inflammasome activation by promoting mtDNA release into the cytosol and acting as a scaffold for immune complex formation. The resulting overproduction of IL-1β and IL-18 leads to chronic inflammation. Furthermore, inflammasome-mediated suppression of mitophagy impairs the clearance of damaged mitochondria, creating a vicious cycle of persistent mitochondrial dysfunction and inflammation.

These mechanisms are particularly relevant to ankylosing spondylitis (AS), where chronic inflammation, mitochondrial dysfunction, and innate immune activation converge. Evidence from previous studies indicates that environmental influences—such as microbiota alterations, infections, and mechanical stress—may precede clinical symptoms by years, contributing to a prolonged preclinical phase characterized by low-grade innate immune activation [31]. However, subclinical inflammation may occur early in AS the frequently cited window of 6 to 10 years likely reflects diagnostic delay rather than a true preclinical phase [77,78]. During this window, mitochondrial stress, mtROS, and cytosolic DNA accumulation may contribute to the early inflammatory signature in AS. In this pre-clinical stage, innate immune activation (involving macrophage, inflammasomes, or interleukins) and low-grade inflammation occur without obvious subclinical and clinical features. Because inflammation has not yet produced irreversible damage, this phase represents the most beneficial time for interventions, if it is properly identified.

In the healthy state, cellular DNA is restricted to the nucleus and in mitochondria (mtDNA), with no DNA structure detectable in cytoplasm under physiological conditions [55]. In AS; however, cytoplasmic dsDNA can accumulate and acts as a DAMP, triggering innate immune activation and promoting systemic inflammation [27]. Human studies have demonstrated increased AIM2 expression in the gut tissue, correlated with increased inflammasome level in peripheral blood mononuclear cells (PBMC), is AS [27]. This pattern is associated with higher levels of IL-1β, IL-18, IL-23A, and increased disease activity score [27]. Together, these findings support the hypothesis that AIM2 contributes to chronic inflammation in AS and may represent part of the innate immune signature of the disease; however, additional studies are needed to confirm these observations. Furthermore, biomechanical stress drives tenocyte cell death by increased transforming growth factor-β (TGFβ) production and the release of DAMPs, which activate inflammasomes and stimulate IL-1β production [79].

Mitochondrial dysfunction thus appears to be a consistent feature of AS. Other inflammatory triggers in AS are related to mitochondrial dysfunction and elevated mitochondrial ROS (mtROS) [80,81]. For example, serum from AS patients induces mtROS production, mitochondrial depolarization, and protein oxidation in mesenchymal stem cells (MSCs) culture [80]. These changes led to the senescence markers (p53, p21, p16) expression, a reduction in mitochondrial membrane potential and ATP production, and triggered inflammation in AS [80]. Interestingly, the mitochondrial-targeted antioxidant (mitoquinone-MitoQ) reverses these effects, highlighting oxidative stress as a major driver of mitochondrial damage in AS [80].

### 2.5. Mechanistic Model Linking AIM2 Activation and Mitochondrial Dysfunction in AS, Crosstalk with Other Innate Sensors

In AS pathology, innate immune activation is supported by mitochondrial dysfunctions. In this light, mitochondrial dysfunction is not only a consequence but also a contributor to chronic inflammation in AS. The AIM2 inflammasome represents one of the key players in these border events, acting through a cascade of tightly interconnected pathways.

In AS, several crosstalk events, such as defective mitophagy, sustained cytokine signaling, and metabolic reprogramming, lead to mitochondrial stress in monocyte/macrophage and T cells. Long-term activation of key pro-inflammatory pathways, including TNF-β and IL-17 signaling pathways, keeps the cells in a chronically pro-inflammatory state. Persistent TNF-α and IL-17 disrupt mitochondrial function by increasing metabolic stress and overproduction of mtROS. The mtROS overproduction causes mitochondrial membrane depolarization and leakage of oxidized mtDNA into the cytosol via mPTP. Both oxidized mtDNA and high ROS levels act as potent ligands of AIM2 inflammasome activation [29,36,37].

**Figure 2:**
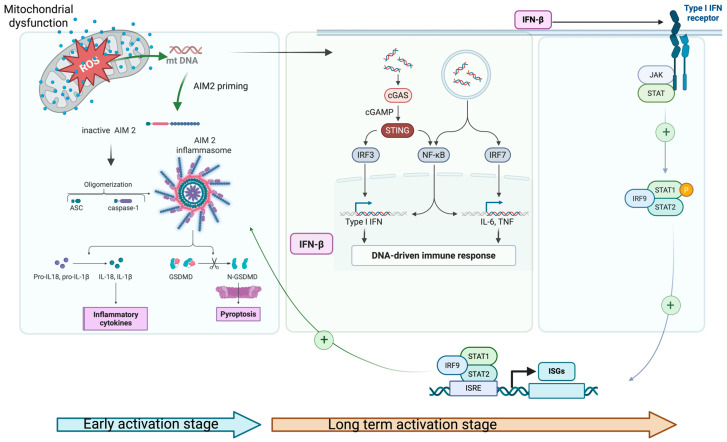
Schematic representation of the interplay between impaired mitophagy, oxidative stress, and AIM2 inflammasome activation; 1. mtROS and ox-mt DNA prime and activate AIM2 inflammasome and c-GAS/STING pathway simultaneously in the early activation stage (blue arrow); 2. INF-β activates the JAK-STAT pathway, increasing AIM2 transcriptional priming through ISGs and supplies pro-IL1-β (NfKB pathway); 3. JAK/STAT signaling pathway product (IL-6/IL-23–STAT3; IFNAR–STAT1/2) stabilizes the inflammatory cell state and maintains mitochondrial stress (via high metabolic/ROS set-point). These events appear in long term activation stage (brown arrow) (Created in 2025 BioRender. b, a. (accessed on 17 November 2025) https://BioRender.com/ygtpswt).

In parallel with inflammasome activation (e.g., NLRP3, AIM2), mtROS and oxidized mtDNA also activate other cytosolic DNA sensors, particularly the interferon signaling arm via cGAS/STING pathway. Once activated, the cGAS/STING pathway induces the type I interferons (IFNs) production, which bind to their receptor and activate the JAK-STAT signaling cascade. JAK-STAT activation leads to the transcription of a broad class of interferon-stimulated genes (ISGs), which in turn alter mitochondrial redox balance and induce metabolic stress. This activation contributes to AIM2 and/or NLPR3 inflammasome priming, increasing pro-IL-1β expression through the NFkB pathway, thereby linking inflammasomes and interferon signaling arms [82,83]. Increased levels of pro-IL-18 and IL-1β further exacerbate mitochondrial damage (feed-back signal), reinforcing a vicious loop of oxidative stress and pro-inflammatory condition (Figure 2).

In AS, ISGs expression pattern represents a hallmark of cGAS/STING–JAK-STAT axis overactivation, even in the context of low circulating IFN levels. These events create a complex picture of chronic inflammation in AS with two phases: a rapid onset phase dominated by inflammasome activation, followed by a long-term phase sustained through cGAS/STING–JAK-STAT axis, which ultimately feeds back to inflammasome priming. This self-maintaining process may explain the persistence of inflammation in AS, even during the cytokine inhibitor therapy. Additionally, activated AIM2 inflammasome increased the K+ efflux, which can indirectly activate NLPR3 inflammasomes, further amplifying the pro-inflammatory signal.

Although the AIM2 inflammasome provides a mechanistic bridge between mitochondrial dysfunction and innate immune activation, we acknowledge that direct human evidence linking AIM2 to AS pathogenesis remains sparse. In an animal model study by Gugino et al., concurrent activation of other inflammasomes (e.g., NLRP3, NLRC4) was observed in the gut tissue of HLA-B27 transgenic rats, supporting the existence of inflammasome-gut microbiota interaction [27]. Moreover, the NLRC4 inflammasome is often co-upregulated with NLPR3 and AIM2 in response to bacterial components (e.g., flagellin or type three secretion systems -TTSS elements), reflecting the bacterial sensing arm of innate immune activation [27]. Notably, NLRP3 inhibition was shown to prevent ileitis and delay arthritis progression in a related disease model, suggesting that the AIM2 inflammasome may act more as an amplifier rather than an initiator hub in AS. However, animal model data consistently support a multi-inflammasome activation pattern in AS, operating in a tissue and trigger-dependent manner [27,84].

This mechanistic concept could explain why biologic therapy that targets downstream cytokines can normalize systemic inflammation but fail to restore mitochondrial function. It further supports the rationale for exploration of combined targeted therapy with cytokine blockers and mitochondria-targeted agents.

## 3. Evidence and Therapeutic Implications

However, direct studies in AS are lacking; broader evidence supports a link between defective mitophagy and mtDNA release, which acts as a DAMP that triggers innate immune activation. To date, it is well established that under inflammatory conditions, damaged mitochondria that are not removed by clearance mechanisms (e.g., mitophagy) can activate the AIM2 inflammasome through mtDNA leakage and mtROS production [85,86,87]. Recent studies have reviewed the role of mitochondrial integrity control and mtDNA signaling in driving chronic inflammation [85,86,87].

While inflammasome activation by mtDNA leakage is well supported in autoimmune and inflammatory diseases, a direct causal link between these processes and AS pathogenesis still requires confirmation through comprehensive clinical studies. Recognizing the contribution of AIM2 and mitochondrial dysfunction to AS pathogenesis provides a new conceptual framework for evaluating both existing biologic therapies and emerging mitochondria-targeted interventions.

Currently, anti-TNF and anti-IL-17 agents represent the first-line biologic disease-modifying antirheumatic drugs (bDMARDs) for refractory AS patients who do not respond to non-steroidal anti-inflammatory therapy (NSAID) or conventional synthetic antirheumatic drugs (csDMARDs) (e.g., methotrexate or sulphasalazyne). Due to their robust efficacy, demonstrated in large-scale clinical studies involving thousands of AS patients, as well as their long-term disease-modifying benefits and safety issues, anti-TNF medication remains the first-choice therapy [88,89,90,91,92,93]. Recently, several new biologic agents have become available as an alternative to anti-TNF therapy, including IL-17 inhibitors and Jak/STAR inhibitors. However, clinical outcomes across trials have been heterogeneous, and some studies have reported conflicting results regarding efficacy and long-term benefit [94,95,96,97,98,99,100,101].

Compared with anti-TNF drugs, which inhibit broad inflammatory signaling networks, IL-17 and JAK/STAT pathway inhibitors exhibit greater target specificity. For instance, monoclonal antibodies against IL-17A (IL-17A mAb) selectively block the IL-23/IL17 axis, targeting the hallmark changes in AS pathogenesis. Interestingly, IL-17 inhibitors proved significant improvement in clinical and functional outcomes, whereas IL-23 inhibitors such as ustekinumab and risankizumab have not shown consistent therapeutic benefit in AS to date [102,103]. These findings suggest that IL-17A signaling in AS may be partly independent of IL-23–mediated pathways.

A recent analysis by Li and colleagues identified 865 clinical trials related to AS in the Informa database, reporting that 66.3% of studies primarily focused on biologic disease-modifying antirheumatic drugs (bDMARDs) aimed at inflammation control. Nevertheless, interest in alternative targeted approaches—including stem cell–based interventions and microbiome-modulating therapies—has grown in recent years [104].

**Figure 3:**
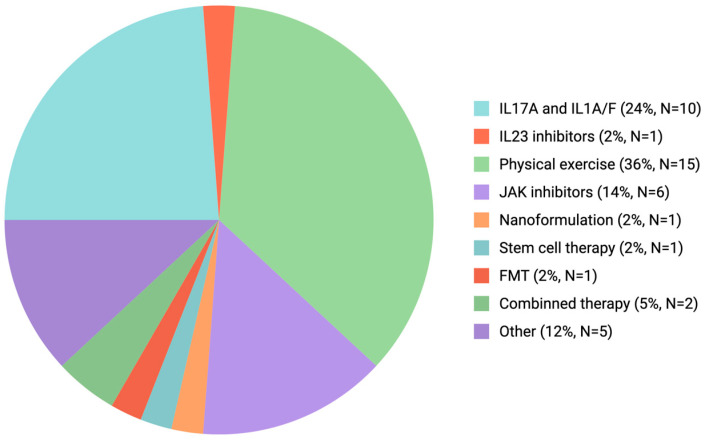
Clinical trials summarizing the therapeutic approach in AS (January 2024–September 2025). Many registered trials focus on biologic disease-modifying antirheumatic drugs (bDMARDs), with IL-17 pathway inhibitors and non-pharmacological interventions such as physical rehabilitation representing the most intensively studied class. Other categories include JAK inhibitors, IL-23 inhibitors, microbiome-targeted therapies (e.g., fecal microbiota transplantation. Abbreviations: AS-ankylosing spondylitis; bDMARDs-biologic disease-modifying antirheumatic drugs; JAK-Janus kinase; IL-interleukin; TNF-tumor necrosis factor).

Our search on databases covering PubMed, Cochrane, Embase, and ClinicalTrials.gov was selected for their extensive indexing of the biomedical and clinical literature. A thorough, multi-database approach was used to find relevant clinical trials that were published between 2024 and September 2025. We employed a comprehensive, multi-database strategy using MeSH terms for “Spondylitis” and “Ankylosing”. The initial search of the database yielded 45 clinical trials, of which 43 were available in English. The distribution and focus of these recent clinical trials are summarized in Figure 3.

A key limitation of the current trial distribution is the incomplete and potentially biased representation of clinical studies in publicly accessible databases, as not all trials are registered or reported with sufficient details. In addition, the heterogeneity of interventions complicates accurate categorization and interpretation of study outcomes. The clinical trial distribution analysis over the past 2 years (see Figure 2) indicates that translational research efforts remain largely focused on a narrow range of therapeutic compounds, with a particular emphasis on IL-17A inhibitors as the most promising emerging agent for AS treatment [103]. Also, rehabilitation strategies, particularly those involving structured physical exercise, have gained increasing scientific and clinical attention as complementary approaches to pharmacologic therapy.

While biologic agents targeting the TNF and IL-17 pathways remain the cornerstone of AS management, the growing exploration of upstream modulators, such as mitochondria-targeting compounds, reflects a broader shift toward more comprehensive and personalized therapeutic strategies.

## 4. Discussion and Future Perspective

Our review highlights the role of AIM2 inflammasome as a cytoplasmic DNA sensor that collects and integrates signals from mitochondrial dysfunction, cellular stress (e.g., ROS), and genomic instability. These findings support the fact that innate immune signals do not simply activate DNA-sensing inflammasomes (e.g., AIM2) pathways but rather prepare them for activation, allowing for a dynamic and context-dependent response. This adaptability highlights the significance of cellular context: depending on the nature of stressors, metabolic state, and balance of cell death-associated gene expression, AIM2 activation can induce various downstream effects, including pyroptosis, apoptosis, or PANoptosis.

The link between genomic instability, micronuclei formation, and AIM2 activation warrants specific attention. Chronic low-grade inflammation is a hallmark of many pathological processes, including neurodegenerative and autoimmune diseases. Strategies that either reduce cytoplasmic DNA accumulation or enhance its clearance may therefore represent attractive therapeutic avenues. Importantly, recent advances in micronuclei biology have revealed that not all micronuclei are equivalent—isolated micronuclei arise in relatively stable genomes, whereas micronuclei clusters are characteristic of unstable genomes. This distinction offers new perspectives on genome-related pathological processes, showing that micronuclei are not only stress-related end products but also active modulators of innate immune signaling.

The emerging concept of PANoptosis—an integrated form of programmed cell death encompassing apoptosis, pyroptosis, and necroptosis—further emphasizes the multifaceted role of AIM2 in diseases. Inflammasomes activation is a key node within this broader cell-death signaling network. In chronic inflammatory diseases such AS, targeting a single pathway may be insufficient to achieve optimal disease control. Instead, strategies aimed at modulating multiple key nodes of PANoptosis or coordinating several cell-death related pathways simultaneously, may yield greater therapeutic efficacy. As current options provide only partial disease control, the AS management is moving toward personalized, targeted therapies. For successful approaches, the new therapeutic opportunities across several directions (e.g., immunological, metabolic, and mitochondrial perspectives) must be explored.

The growing body of literature on AS therapeutics reflects this diversification of interventions tested in clinical trials. It can be explained by the fact that current options for AS management often yield incomplete responses. Also, patients may respond differently to various drugs, which makes it difficult to select the optimal therapy for better disease outcome control.

Despite the fact that mitochondrial biology is increasingly recognized as a key driver of AS, research on mitochondria-targeted therapy remains largely experimental [105]. Preclinical studies have identified several mitochondria-targeted compounds with encouraging results that warrant future translational investigation. For example, N-acetyl cysteine (NAC), a ROS scavenger, has demonstrated efficacy in reducing oxidative stress and inflammatory responses in autoimmune diseases, including AS [106]. A study by Navid and colleagues on HLA-B27+ macrophages concluded that oxidative stress contributes to ER stress–driven inflammatory responses. ROS scavenging by NAC mitigates unfolding protein response activation and cytokine production, suggesting that targeting oxidative stress pathways may represent a viable therapeutic approach in AS [106].

Other promising preclinical strategies include agents that enhance mitophagy, such as rapamycin, an mTOR pathway inhibitor, which reduced IL-17A and TNF-α levels in rat models [107].

Similarly, NAD^+^-a key redox coenzyme essential for energy metabolism, is frequently depleted in mitochondrial dysfunction associated with chronic inflammation [108]. Cellular NAD^+^ balance depends on the rate of synthesis and consumption [109]. An imbalance in NAD^+^ metabolism due to overconsumption or reduced synthesis often drives NAD^+^ depletion under inflammatory conditions [109]. Enhancing NAD^+^ availability can promote mitophagy, reduce mtDNA leakage, and restore mitochondrial clearance. Consequently, lowering mtDNA leakage and mitochondrial ROS can attenuate AIM2 inflammasome activation [110,111,112].

In arthritis models, NAD^+^ supplementation has been shown to reduce oxidative stress, increase intracellular NAD^+^ levels, suppress IL-1β production, and improve inflammation control [113]. To date, NAD+ boosting compounds have not yet been tested or used in AS models; these findings provide a mechanistic rationale for developing further protocols to evaluate their efficacy in AS models.

Other promising agents, such as Urolithin A (a benzo-coumarin derivative), a postbiotic metabolite released by gut microbiota, have demonstrated anti-inflammatory and antioxidant effects, primarily through enhancing mitophagy and reducing IL-1β and TNF-α production [114,115,116,117]. Such compounds must be further studied in clinical trials to prove the benefits in AS patients. To date, no available clinical trials have specifically evaluated the role of Urolithin A in AS, but some clinical trials have proved safety and good tolerability in humans [118,119]. Given its favorable safety profile and beneficial effects on cellular processes, Urolithin A remains a promising therapeutic candidate for future studies in AS trials.

Over the past two decades, inflammasome research has established these complexes as key regulators of innate immunity and chronic inflammation. Among them, AIM2 stands out as a unique DNA sensor that links cytosolic dsDNA, mitochondrial dysfunction, and uncontrolled inflammatory signaling. Evidence suggests that AIM2 activation drives pyroptosis and pro-inflammatory cytokine release and contributes to PANoptosis, thereby integrating multiple cell-death pathways.

In AS, accumulating evidence indicates that AIM2 inflammasome activation, driven by mtROS and mtDNA leakage, represents an important mechanism linking environmental triggers, metabolic stress, and genetic susceptibility to chronic inflammation. However, while anti-TNF and anti-IL-17 therapies provide substantial clinical benefit, they do not target the upstream processes of mitochondrial dysfunction and innate immune overactivation.

In this light, future research should aim to solve several knowledge gaps: first, better defining the context-dependent roles of AIM2 activation in protective versus pathological states; second, elucidating how micronuclei accumulation triggers AIM2 activation in AS; third, dissecting the interplay between mitochondrial dysfunction, dsDNA sensing, and cell-death mechanisms using disease-relevant models.

Strategies designed to restore mitochondrial homeostasis—including ROS scavengers, mitophagy enhancers, NAD^+^ boosters, and postbiotic compounds such as Urolithin A—represent promising avenues for translational research. Nevertheless, direct evidence in AS remains limited, and well-designed preclinical and clinical studies are required to validate AIM2 as a viable therapeutic target.

Currently, human single-cell and transcriptomic datasets have identified distinct immune signatures in AS but provide only limited insight into the mechanistic link between mitochondrial dysfunction, mtDNA leakage/micronuclei, and AIM2 activation [120,121,122,123,124]. Further analysis of available scRNA-seq and transcriptomic AS datasets could determine whether ISGs are upregulated in specific immune cell populations—particularly monocytes, macrophages, or dendritic cells. Such findings would support the hypothesis that the DNA-sensing pathway (via AIM2/IFI16) and mitochondrial stress converge to drive AS inflammation. However, any identified pattern must be further validated in “in vivo” AS models.

Despite significant progress in understanding inflammasome biology and the development of new effective therapies for AS, several limitations remain. Some promising preclinical findings have failed to translate into clinical use for AS. This may be due to the preclinical gaps, particularly to the mechanistic insights into AIM2 activation and its connection to mitochondrial dysfunction, which are often derived from “in vitro” studies or non-AS disease models that fail to fully capture disease complexity. Direct evidence from in vivo AS models remains scarce, thereby limiting the translational relevance of current findings.

By positioning AIM2 inflammasome activation within the broader context of mitochondrial biology, DNA damage, and cell-death signaling, this review supports a paradigm shift in AS treatment: successful management of complex diseases such as AS requires modulation of upstream danger signals and cellular stress responses. Novel upstream targets—including integrated signaling pathways such as inflammasomes, mitochondrial dysfunction, or DNA clearance pathways—remain largely unexplored in human trials.

Moreover, many early trials were small, open-label, or uncontrolled, limiting their statistical power and reproducibility. Incomplete or delayed reporting further restricts the strength of the current evidence base, and longitudinal data remain scarce. Most available trials assess only short- to mid-term outcomes, with long-term efficacy and safety data still lacking, particularly for newer agents such as JAK inhibitors or dual IL-17A/F inhibitors.

Taken together, understanding the intricate interplay between mitochondrial biology, dsDNA-driven AIM2 inflammasome activation, and chronic inflammation provides a framework for more precise and personalized therapeutic interventions in AS. Addressing these interrelationships will help bridge the gap between clinical manifestation and underlying molecular mechanisms, ultimately improving patient outcomes.

## 5. Conclusions

AIM2 inflammasome is gaining increasing recognition for its role in autoimmunity. Its activation integrates key cellular processes, including mitochondrial dysfunction and cytosolic DNA sensing, that contribute to chronic inflammation in AS. While current bDMARDs primarily target downstream cytokine signaling, emerging strategies aimed at restoring mitochondrial homeostasis, such as ROS scavengers or mitophagy enhancers, hold promise for future AS management. Targeting AIM2-driven pathways together with mitochondria-targeted interventions and cytokine modulation may open the gate for new personalized, multimodal, and integrated therapeutic approaches for AS.

## Abbreviations

The following abbreviations are used in this manuscript:

AS	Ankylosing Spondylitis
AIM2	Absent in melanoma 2
NLPR3	NLR family pyrin domain-containing protein 3
NLPR1	NLR family pyrin domain-containing protein 1
dsDNA	double-stranded Deoxyribonucleic Acid
mt-DNA	Mitochondrial DNA
PAMP	Pathogen-associated molecules
DAMP	Molecules associated with danger
HAMP	Molecular processes that alter homeostasis
ISGs	Interferon-stimulated genes
DNase	Deoxyribonuclease
TREX1	Three prime repair exonuclease 1
TNF	tumor necrosis factor
IL-17	Interleukin-17
JAK	Kinaza Janus
Casp 1	Caspase 1
IL-1β	Interleukin 1beta
IL-18	Interleukin 18
PYD	Pyrin domain
CARD	CARD domain
cDNA	cytosolic DNA
NLRC4	NLR family CARD domain-containing protein 4
ASC	Speck-like Protein associated with apoptosis that continues a CARD domain
NLR	Nucleotide-binding domain, Leucine-rich Repeat
ALR	AIM2-like receptor
TNF α	Tumor necrosis factor alfa
INFγ	Gamma Interferon
ROS	Reactive oxygen species
NOS	Reactive nitrogen species
IFI16	Gamma-interferon-inducible protein 16
IFIX	pyrin and HIN-containing domain protein 1
MNDA	myeloid cell nuclear differentiation antigen
HIN200	hematopoietic interferon-inducible nuclear protein with a 200-amino acid repeat domain
NFkB	Nuclear factor kappa-light-chain-enhancer of activatd B cells
ATP	Adenosine triphosphate
GSDMD	Gasdermin D
LPS	Lipopolysaccharide
GMP-AMP	Guanosine monophosphate-adenosine monophosphate
cGAS-STING	GMP-AMP cyclic synthesis-stimulator of interferon genes
mPTP	Mitochondrial permeability transition pore
HLA B27	Antigen leukocyte human B27
PBMC	Mononuclear cells in peripheral blood
IL 23A	Interleukin 23A
TGFβ	Transforming growth factor beta
mtROS	Mitochondrial ROS
MSC	Mesenchymal stem cells
NSAIDs	Non-steroidal anti-inflammatory drugs
bDMARDs	Biological Disease-Modifying Antirheumatic Drugs
csDMARDs	Conventional Synthetic Disease-Modifying Antirheumatic Drugs
scRNA-seq	single-cell RNA sequencing
JAK/STATE	Janus Kinase Pathway/Transcription Protein Activator
IL17AmAb	Interleukine 17 monoclonal antibodies
MeSH	Medical Subiect Headings
NAC	N acetyl cysteine
mGATE	mTOR gate
NAD	Nicotinamide adenine dinucleotide
IL-1	Interleukin-1
